# Localization of the cannabinoid CB1 receptor and the 2-AG synthesizing (DAGLα) and degrading (MAGL, FAAH) enzymes in cells expressing the Ca^2+^-binding proteins calbindin, calretinin, and parvalbumin in the adult rat hippocampus

**DOI:** 10.3389/fnana.2014.00056

**Published:** 2014-06-27

**Authors:** Patricia Rivera, Sergio Arrabal, Manuel Cifuentes, Jesús M. Grondona, Margarita Pérez-Martín, Leticia Rubio, Antonio Vargas, Antonia Serrano, Francisco J. Pavón, Juan Suárez, Fernando Rodríguez de Fonseca

**Affiliations:** ^1^Laboratorio de Investigación, Instituto de Investigación Biomédica (IBIMA), Universidad de Málaga-Hospital Regional Universitario de Málaga (UGC Salud Mental)Málaga, Spain; ^2^CIBER OBN, Instituto de Salud Carlos III, Ministerio de Ciencia e InnovaciónMadrid, Spain; ^3^Departamento de Biología Celular, Genética y Fisiología, Facultad de Ciencias, Instituto de Investigación Biomédica (IBIMA), Universidad de MálagaMálaga, Spain; ^4^CIBER BBN, Instituto de Salud Carlos III, Ministerio de Ciencia e InnovaciónMadrid, Spain; ^5^Departamento de Anatomía y Medicina Legal, Facultad de Medicina, Universidad de MálagaMálaga, Spain

**Keywords:** cannabinoid receptor, 2-arachidonoylglycerol, calcium-binding protein, hippocampus, rat, immunohistochemistry, confocal microscopy

## Abstract

The retrograde suppression of the synaptic transmission by the endocannabinoid *sn-2-arachidonoylglycerol* (2-AG) is mediated by the cannabinoid CB1 receptors and requires the elevation of intracellular Ca^2+^ and the activation of specific 2-AG synthesizing (i.e., DAGLα) enzymes. However, the anatomical organization of the neuronal substrates that express 2-AG/CB_1_ signaling system-related molecules associated with selective Ca^2+^-binding proteins (CaBPs) is still unknown. For this purpose, we used double-label immunofluorescence and confocal laser scanning microscopy for the characterization of the expression of the 2-AG/CB_1_ signaling system (CB_1_ receptor, DAGLα, MAGL, and FAAH) and the CaBPs calbindin D28k, calretinin, and parvalbumin in the rat hippocampus. CB_1_, DAGLα, and MAGL labeling was mainly localized in fibers and neuropil, which were differentially organized depending on the hippocampal CaBPs-expressing cells. CB^+^_1_ fiber terminals localized in all hippocampal principal cell layers were tightly attached to calbindin^+^ cells (granular and pyramidal neurons), and calretinin^+^ and parvalbumin^+^ interneurons. DAGLα neuropil labeling was selectively found surrounding calbindin^+^ principal cells in the dentate gyrus and CA1, and in the calretinin^+^ and parvalbumin^+^ interneurons in the pyramidal cell layers of the CA1/3 fields. MAGL^+^ terminals were only observed around CA1 calbindin^+^ pyramidal cells, CA1/3 calretinin^+^ interneurons and CA3 parvalbumin^+^ interneurons localized in the pyramidal cell layers. Interestingly, calbindin^+^ pyramidal cells expressed FAAH specifically in the CA1 field. The identification of anatomically related-neuronal substrates that expressed 2-AG/CB_1_ signaling system and selective CaBPs should be considered when analyzing the cannabinoid signaling associated with hippocampal functions.

## Introduction

**Figure 1:**
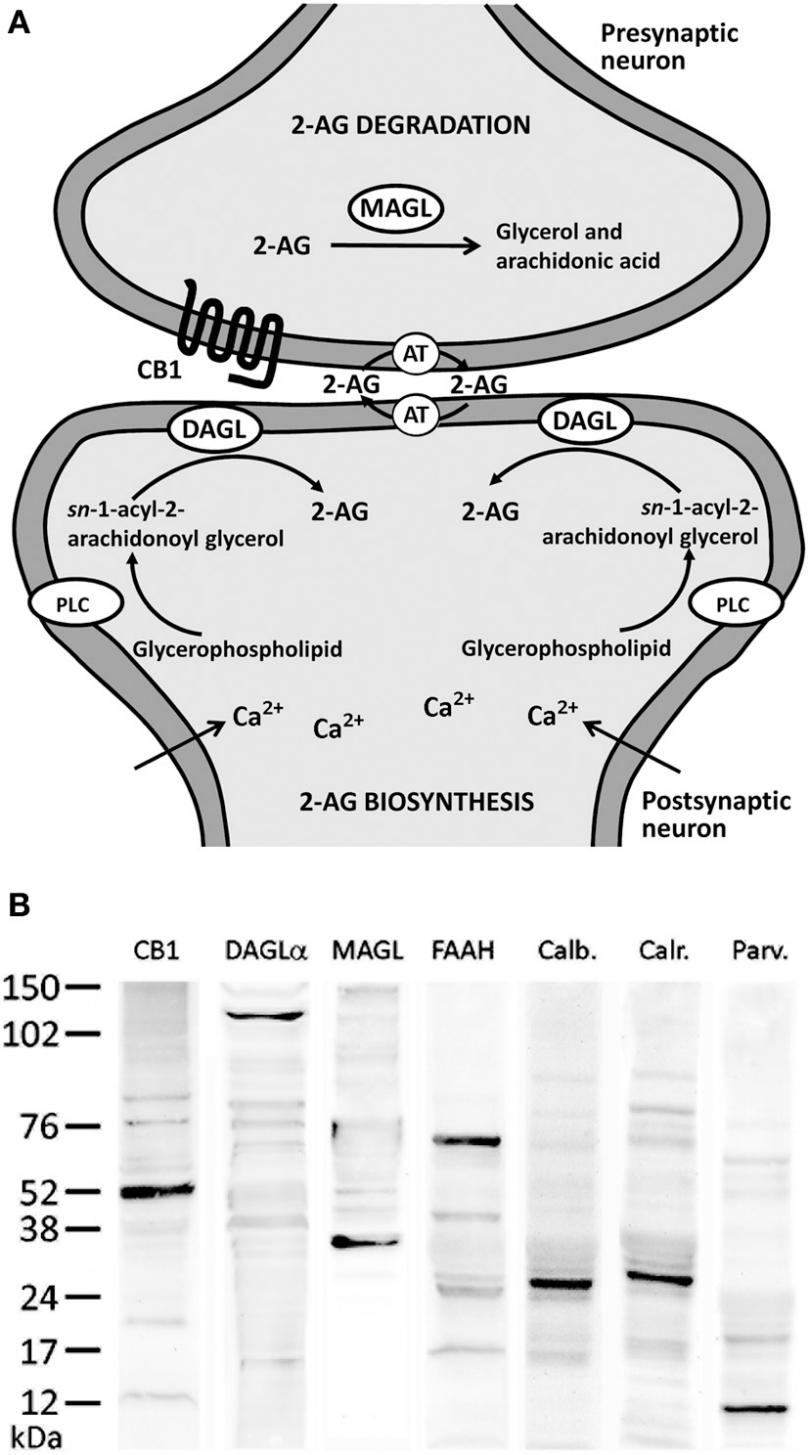
**(A)** Major biochemical pathway for 2-AG signaling system. Postsynaptic calcium influx and the activation of metabolic receptors coupled to phosphotidyl-inositol-specific phospholipase C (PLC) and diacylglycerol lipase (DAGL) pathway lead to increases in 2-AG production. 2-AG signaling includes uptake into cells mediated by an unknown transporter (AT) and hydrolysis by specific enzymatic systems, such as MAGL. **(B)** Western blots of protein extracts from rat hippocampus showing CB_1_ immunostaining as a prominent band at about 52 kDa. Immunoblots for DAGLα, MAGL, and FAAH also revealed a single band with molecular masses of 120, 35, and 63 kDa, respectively. Analysis of calbindin D28k, calretinin, and parvalbumin immunoblottings confirmed expected bands of 28, 29, and 10 kDa, respectively.

*ns*-2-arachidonoylglycerol (2-AG), one of the endogenous ligands for cannabinoid receptors, regulates synaptic transmission in the nervous system by acting as a retrograde inhibitory signal of excitatory/inhibitory synapses (Katona and Freund, 2008; Stella, 2009, see Figure 1A for summary). This retrograde signaling requires the participation of the divalent cation calcium (Ca^2+^), production/release of 2-AG and calcium mobilization leading downstream cannabinoid receptor signaling that ultimately lead to the inhibition of presynaptic neurotransmitter release (D'Amico et al., 2004; Di et al., 2005; Katona and Freund, 2008; see Sugiura et al., 2006 for review). For instance, the postsynaptic endocannabinoid release can be triggered in response to specific stimuli that mobilizes Ca^2+^, including either depolarization-induced postsynaptic Ca^2+^ elevation or activation of postsynaptic G_q/11_-coupled receptors (i.e., mGluR1-phospholipase C β pathway) with or without Ca^2+^ elevation (Kondo et al., 1998; Maejima et al., 2005; Ohno-Shosaku et al., 2005). Effective production of 2-AG from diacylglycerol (DAG) is demonstrated by combined weak mGluR1-PLC β1/4 cascade activation and Ca^2+^ elevation to a submicromolar range (Maejima et al., 2001, 2005; Hashimotodani et al., 2005; Ohno-Shosaku et al., 2005).

The retrograde signaling mediated by 2-AG is determined by the balance between the production and clearance of 2-AG. The primary route of 2-AG synthesis in the nervous system has been proposed to occur via hydrolysis of DAG by the *sn*-1-specific diacylglycerol lipase alpha (DAGLα) in a Ca^2+^-dependent manner (Bisogno et al., 2003; Gao et al., 2010; Tanimura et al., 2010). DAGLα, essentially targeted to postsynaptic spines, enables efficient 2-AG production for a swift modulation onto nearby presynaptic terminals expressing CB_1_ receptor (Yoshida et al., 2006). Thus, intracellular Ca^2+^ elevation-induced DAGLα activation is essential for the endocannabinoid-mediated synaptic plasticity (Gao et al., 2010; Tanimura et al., 2010). Monoglyceride lipase (MAGL) and fatty acid amide hydrolase (FAAH) have been identified as degrading enzymes of endogenous cannabinoids (Di Marzo et al., 1998, 1999; Goparaju et al., 1998, 1999). Breakdown of 2-AG has been mostly attributed to MAGL (Dinh et al., 2002; Blankman et al., 2007; Chanda et al., 2010; Schlosburg et al., 2010), whereas FAAH is the main anandamide-metabolizing enzyme (Cravatt et al., 2001; Lichtman et al., 2002). However, FAAH and other uncharacterized enzymes (ABHD6 and ABHD12) also showed 2-AG hydrolase activity (Di Marzo et al., 1998; Goparaju et al., 1998; Blankman et al., 2007). MAGL and FAAH displayed distinct subcellular compartmentalization (Gulyás et al., 2004). Thus, MAGL is primarily a presynaptic enzyme found in axon terminals (Dinh et al., 2002; Gulyás et al., 2004), whereas FAAH is a postsynaptic enzyme associated with membranes of cytoplasmic organelles known to store Ca^2+^ localized in somata and dendrites (Tsou et al., 1998b; Egertová et al., 2003; Gulyás et al., 2004).

The subcellular localization of molecules involved in 2-AG production—including DAGLα, mGluRs, G_p/11_ protein and PLCβ s—in dendritic spines of glutamatergic excitatory synapses is highly associated with intracellular Ca^2+^ stores in a synapse-specific manner. This has been also demonstrated for the CB1 receptor and the degrading enzyme MAGL in nerve terminals of inhibitory synapses (Katona et al., 2006; Kawamura et al., 2006; Katona and Freund, 2008; Uchigashima et al., 2011). Regarding this issue, calcium-binding proteins (CaBPs) play an important role as intracellular Ca^2+^ buffers and sensors in mediating Ca^2+^-dependent events such as synaptic transmission and axonal transport (Nakamura et al., 1980). Several CaBPs, including calbindin D28k, calretinin, and parvalbumin, have been found in high concentrations in the brain (Baimbridge et al., 1982; Garcia-Segura et al., 1984). These CaBPs usually correlate with the neurotransmitter content and cell morphology, distribution and function (Baimbridge et al., 1982; Celio, 1990; Gulyás et al., 1991), being used to classify neurons into specific subpopulations. For instance, hippocampal non-pyramidal cells containing calretinin and parvalbumin are usually GABAergic neurons (Kosaka et al., 1987; Miettinen et al., 1992; Wouterlood et al., 2001). Moreover, it was demonstrated that parvalbumin-positive CA1 interneurons are required for spatial working but not for reference memory (Murray et al., 2011) and calbindin-positive granule cells of dentate gyrus contribute to verbal memory impairments in temporal lobe epilepsy (Karádi et al., 2012). All these cognitive processes are also mediated by the endocannabinoid system in the hippocampus, making necessary the identification of the cellular networks involved (Riedel and Davies, 2005; Puighermanal et al., 2012).

Since CaBPs buffer intracellular Ca^2+^ levels, they can influence both synthesis/release and CB_1_ signaling in the hippocampus and, as a consequence, the synaptic plasticity processes. This hypothesis supports the need for the identification of the neural substrates that express the CaBPs and the 2-AG/CB1 signaling system. In the present study, we described and systematically characterized the neuronal structures that co-express the 2-AG/CB1 signaling system (CB_1_ receptor, DAGLα, MAGL, and FAAH) with the CaBPs calbindin D28k, calretinin, and parvalbumin in the rat hippocampus. For this purpose, we used double-label immunofluorescence and confocal laser (spectral) scanning microscopy.

## Materials and methods

### Animals

Adult male Wistar rats (*n* = 5), weighing approximately 250 g and 10–12 weeks old (Charles River Laboratories, Barcelona, Spain), were used in this study. The animals were kept in standard conditions (Servicio de Estabulario, Facultad de Medicina, Universidad de Málaga) at 20 ± 2°C room temperature, 40 ± 5% relative humidity and a photoperiod of 12L:12D; the rats were given free access to food and water. All experimental animal procedures were performed in compliance with the European Communities directive 86/609/ECC and Spanish legislation (BOE 252/34367-91, 2005) regulating animal research.

### Tissue processing

The animals were anesthetized with sodium pentobarbital (50 mg/kg, i.p.) and transcardially perfused with 0.1 M phosphate-buffered saline (PBS; pH 7.3), followed by 4% formaldehyde in PBS. The brains were dissected and incubated in the same fixative solution overnight at 4°C and then cryoprotected in 0.1 M phosphate-buffered saline pH 7.3 (PBS) containing 30% sucrose and 0.01% sodium azide (NaN_3_) for 48 h. Then, the brains were cut into 30-μm-thick transverse sections using a sliding microtome. The sections were stored at 4°C in PBS with 0.002% (*w*/*v*) NaN_3_ until immunohistochemistry analysis.

### Immunohistochemistry

For the analysis of the immunohistochemical expression of CB_1_, DAGLα, MAGL, FAAH, and the Ca^2+^-binding proteins (calbindin, calretinin, and parvalbumin) in the hippocampus, free-floating 30-μm-thick coronal sections from −3.00 to −4.80 mm Bregma levels (Paxinos and Watson, 2007). The sections were first washed several times with 0.1 M PBS (pH 7.3) to remove the NaN_3_ and were incubated in H_2_O containing 50 mM sodium citrate (pH 6) for 30 min at 80°C, followed by several washes in 0.1 M PBS (pH 7.3). Then, the sections were incubated in a solution of 3% H_2_O_2_ and 10% methanol in 0.1 M PBS for 20 min at room temperature in the dark to inactivate the endogenous peroxidase, followed by washes in PBS. The sections were then blocked with 10% donkey or goat serum in PBS containing 0.1% NaN_3_ and 0.2% Triton X-100 and incubated with a primary antibody overnight at room temperature (for details regarding the antibodies used, see Tables 1, 2).

**Table 1:** **Primary antibodies used**.

**Antigen**	**Immunogen**	**Manufacturing details**	**Dilution**	**References**
CB1	Mouse CB1, C-terminal 31 aa (NM007726)	Frontier Institute	1:200	Uchigashima et al., 2007
		Polyclonal antibody		
		Developed in rabbit		
		Code No.: CB1-Rb-Af380-1		
		Lot. No.: Not provided		
DAGLα	A 16-aa peptide from the C-terminal region (CGASPTKQDDLVISAR)	Developed by our group	1:250	Yoshida et al., 2006;
		Polyclonal antibody		Suárez et al., 2011
		Developed in rabbit		
MAGL	Mouse MGL, 1-35 aa (NM_011844)	Frontier Institute	1:200	Uchigashima et al., 2011
		Polyclonal antibody		
		Developed in rabbit		
		Code No.: MGL-Rb-Af200		
		Lot. No.: Not provided		
FAAH	Synthetic peptide from rat FAAH, aa 561-579 (CLRFMREVEQLMTPQKQPS)	Cayman	1:200	Tsou et al., 1998b;
		Polyclonal antibody		Gulyás et al., 2004
		Developed in rabbit		
		Code No.: 101600		
		Lot. No.: 157878		
Calbindin	Calbindin D28k purified from chicken gut:	Swant	1:500	Celio, 1990;
	MTAETHLQGVEISAAQFFEIWHHYDSDG NGYMDGKELQNFIQELQQARKKAGLDL TPEMKAFVDQYGKATDGKIGIVELAQVL PTEENFLLFFRCQQLKSSEDFMQTWRKY DSDHSGFIDSEELKSFLKDLLQKANKQIE DSKLTEYTEIMLRMFDANNDGKLELTEL ARLLPVQENFLIKFQGVKMCAKEFNKAF EMYDQDGNGYIDENELDALLKDLCEKN KKELDINNLATYKKSIMALSDGGKLYRA ELALILCAEEN	Monoclonal IgG antibody		Rüttimann et al., 2004
		Produced in mouse myeloma cells		
		Code No.: 300		
		Lot. No.: 07 (F)		
Calretinin	Recombinant human calretinin 22k (epitope within the first 4 EF-hands domains):	Swant	1:500	Zimmermann and Schwaller, 2002;
	MAGPQQQPPYLHLAELTASQFLEIWKHF DADGNGYIEGKELENFFQELEKARKGSG MMSKSDNFGEKMKEFMQKYDKNSDGK IEMAELAQILPTEENFLLCFRQHVGSSAE FMEAWRKYDTDRSGYIEANELKGFLSDL LKKANRPYDEPKLQEYTQTILRMFDLNG DGKLGLSEMSRLLPVQENFLLKFQGMKL TSEEFNAIFTFYDKDRSGYIDEHELDALL KDLYEKNKKEINIQQLTNYRKSVMSLAE AGKLYRKDLEIVLCSEPPM	Monoclonal antibody		Rüttimann et al., 2004
		Developed in mouse		
		Code No.: 6B3		
		Lot. No.: 010399		
Parvalbumin	Parvalbumin purified from carp muscles:	Swant	1:500	Celio, 1986;
	MAFAGILNDADITAALQGCQAADSFDY KSFFAKVGLSAKTPDDIKKAFAVIDQDK SGFIEEDELKLFLQNFSAGARALTDAETK AFLKAGDSDGDGKIGVDEFAALVKA	Monoclonal IgG antibody		Bouilleret et al., 2000
		Produced in mouse myeloma cells		
		Code No. 235		
		Lot. No.: 10-11 (F)		

**Table 2:** **Secondary antibodies used**.

**Antigen**	**Produced in**	**Conjugate to**	**Manufacturing details**	**Dilution**
Anti-rabbit IgG	Donkey	Biotin	GE Healthcare	1:500
			Code No.: RPN1004	
			Lot. No.: 5356499	
Anti-mouse IgG	Goat	Biotin	SIGMA	1:500
			Code No.: B 7264	
			Lot. No.: 125K6063	
Anti-rabbit IgG	Donkey	Cy3 bis-NHS ester	Jackson ImmunoResearch	1:300
			Code No.: 711-166-152	
			Lot. No.: 101675	
Anti-mouse IgG	Goat	Fluorescein Isothiocyanate (FITC)	SIGMA	1:300
			Code No.: F2012	
			Lot. No.: 107K6058	

The following day, the sections were washed in PBS and incubated in a biotinylated secondary antibody diluted 1:500 for 1 h (Table 2). The sections were washed again in PBS, and incubated with a 1:2000 dilution of ExtrAvidin peroxidase (Sigma, St. Louis, MO) for 1 h. After several washes, immunolabeling was revealed by exposure to 0.05% diaminobenzidine (DAB; Sigma), 0.05% nickel ammonium sulfate and 0.03% H_2_O_2_ in PBS. After several washes in PBS, the sections were mounted on slides treated with poly-l-lysine solution (Sigma), air-dried, dehydrated in ethanol, cleared with xylene, and coverslipped with Eukitt mounting medium (Kindler GmBH & Co, Freiburg, Germany). Digital high-resolution photomicrographs of the rat brain were taken under the same conditions of light and brightness/contrast by an Olympus BX41 microscope equipped with an Olympus DP70 digital camera (Olympus Europa GmbH, Hamburg, Germany).

### Double immunofluorescence

Hippocampal sections were pretreated as described above and incubated overnight at room temperature with a cocktail of primary antibodies (Table 1). After washing in 0.1 M PBS (pH 7.3), the sections were incubated at room temperature with a cocktail of fluorescent secondary antibodies (Table 2) for 2 h. For epifluorescence analysis, digital high-resolution microphotographs were taken with an Olympus BX41 fluorescence microscope equipped with an Olympus DP70 digital camera (Olympus). For a more detailed analysis (Rivera et al., 2014), the sections that were doubly labeled were visualized with a confocal laser (spectral) scanning microscope (Leica TCS NT; Leica Microsystems) equipped with a 561 nm DPM laser (argon 30%) and a 63× objective (HCX PL APO CS 63.0 × 1.40 OIL UV). The numerical aperture was 1.40. The emission filter settings were 504–545 nm for PMT2 (green) and 570–630 nm for PMT3 (red). The images were acquired in sequential mode with a frame average of 3. Depending of the level of zoom used in each image, the XY voxel size ranged from 240.5 nm (zoom = 1) to 30.2 nm. The pinhole (airy) was 1. The section thickness (Z) was 772 nm and Z-stepping increment was 130 nm. Thus, we could discriminate the labeling of those structures whose size was larger than the image resolution. Settings of light and brightness/contrast were adjusted by using the Leica LAS AF Lite imaging software.

### Antibody specific and controls

We performed Western blot analyses to demonstrate that the CB_1_, DAGLα, MAGL, FAAH, calbindin, calretinin, and parvalbumin antibodies recognized the corresponding antigen in the rat hippocampus. To perform Western blot analysis, we used fresh tissue from Wistar male rats. The animals were sacrificed using 2,2,2-tribromoethanol (Fluka, Steinheim, Germany) and the hippocampi were immediately isolated, snap frozen in liquid nitrogen and stored at −80°C until use. Protein extracts of rat hippocampi were prepared in RIPA buffer (50 mM Tris ClH pH 7.4, 150 mM NaCl, 0.25% NaDOC, 1% triton X100, 1 mM EDTA, 10% aprotinin) using a homogenizer. After 2 h of incubation in agitation at 4°C, the homogenate was centrifuged at 20800 *g* for 20 min at 4°C, and the supernatant was collected.

For immunoblot analysis, equivalent amounts of protein extracts (75 μg) were separated by a 4–20% precast polyacrylamide gel (Criterion™ TGX™ Precast Gel, Bio-Rad, cat. no. 567–1093), electroblotted onto nitrocellulose membranes and stained with Ponceau red to ensure equal loading. The blots were first incubated with a blocking buffer containing 2% bovine serum albumin (Merck) in PBS and 0.1% Tween 20 at room temperature for 1 h. Then, each blotted membrane lane was incubated separately with the specific CB_1_ (1:200), DAGLα (1:100), MAGL (1:200), FAAH (1:200), calbindin D28k (1:500), calretinin (1:1000), and parvalbumin (1:1000) antibodies. Peroxidase-conjugated goat anti-rabbit, goat anti-mouse, and goat anti-guinea pig antibodies (dilution 1:2000; Promega, Madison, WI, USA) were added for 1 h at room temperature. The specific protein bands were visualized using the enhanced chemiluminiscence technique (ECL, Amersham) and Auto-Biochemi Imaging System (LTF Labortechnik GmbH, Wasserburg/Bodensee, Gemany). Western blot analysis showed that each primary antibody detected a protein of the expected molecular size (Figure 1B).

Additional control experiments were carried out in previous studies for antibody specificity. Hippocampus of wild-type mouse was compared to CB1 (Uchigashima et al., 2007), DAGLα (Yoshida et al., 2006; Suárez et al., 2011), MAGL (Uchigashima et al., 2011), and FAAH (Gulyás et al., 2004) knock-out mice. These studies showed that immunostaining was almost completely absent in the respective knock-out mouse hippocampus when compared to wild-type mouse hippocampus (see references in Table 1 for further information).

Calbindin D28k, calretinin and parvalbumin antibodies were also evaluated for specificity and potency (see references in Table 1) using several methods: (a) by indirect immunofluorescent or immunoperoxidase labeling, as well as biotin-avidin labeling, of 4% paraformaldehyde fixed brains; (b) by immunoenzymatic labeling of immunoblots; (c) by radioimmunoassay; or (d) by immunohistochemistry on brain tissue of calbindin knock-out mice, calretinin knock-out mice, and parvalbumin knock-out mice, respectively.

## Results

In the present study, we first analyzed the distribution and, secondly, the co-expression of either the CB_1_ receptor or the enzymes DAGLα, MAGL, and FAAH with the CaBPs calbindin, calretinin, and parvalbumin in the rat hippocampus. The intensity of the immunoreactivity for each antibody used was similar in all brains analyzed for the present study. Previously, we performed Western blot analysis to ensure that CB_1_ receptor, DAGLα, MAGL, FAAH, calbindin, calretinin, and parvalbumin antibodies recognize the corresponding antigens in the rat hippocampus (Figure 1B). Thus, Western blot analyses of protein extracts from rat hippocampus revealed CB_1_ immunostaining as a prominent band at approximately 52 kDa. Immunoblots for DAGLα, MAGL, and FAAH also revealed a single band with molecular masses of 120, 35, and 63 kDa, respectively. Analysis of calbindin D28k, calretinin, and parvalbumin immunoblottings confirmed expected bands of 28, 29, and 10 kDa, respectively (Figure 1B).

### Distribution of CB1, DAGLα, MAGL, FAAH in the adult rat hippocampus

**Figure 2:**
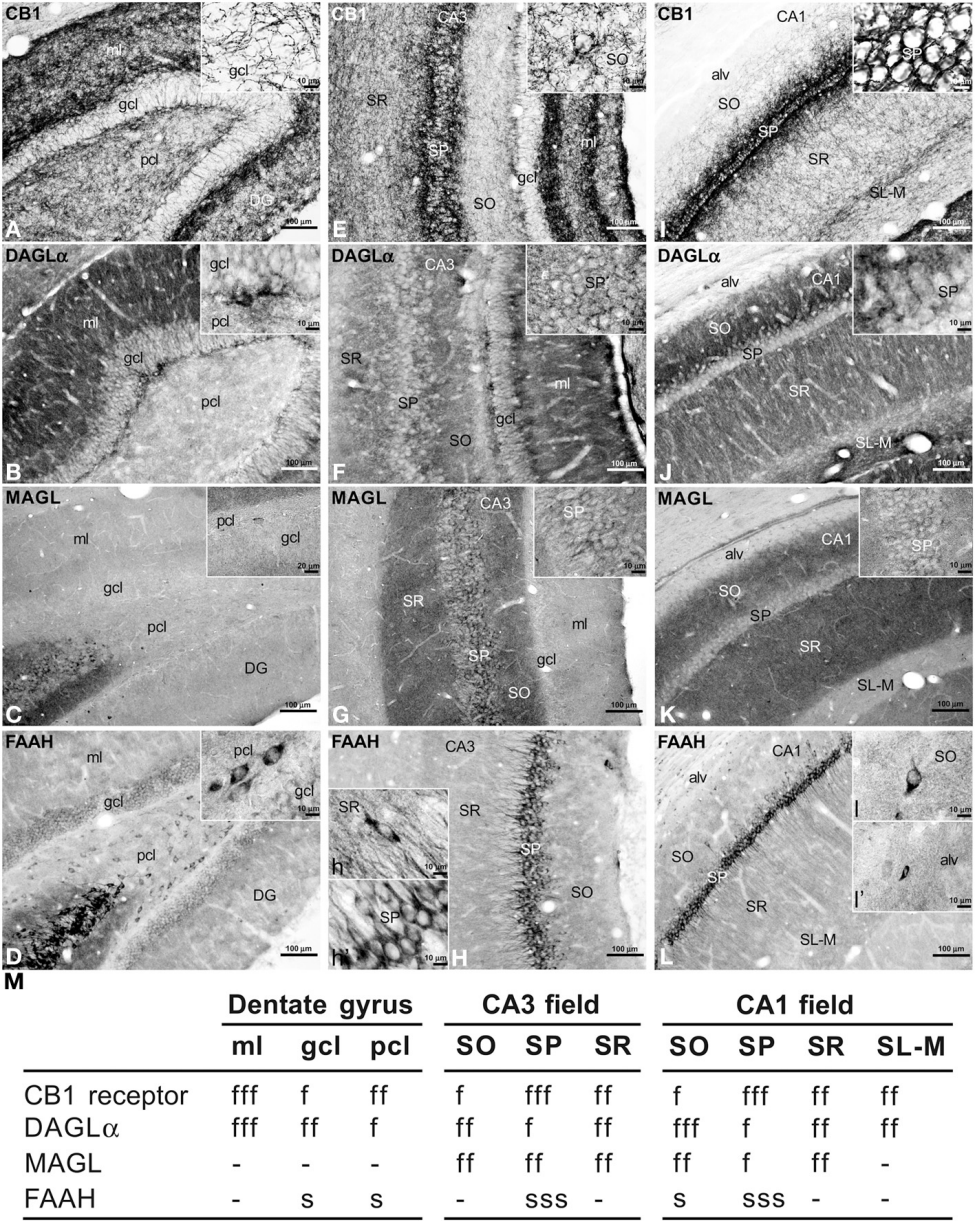
**Immunohistochemical expression of CB_1_ (A,E,I), DAGLα (B,F,J), MAGL (C,G,K), FAAH (D,H,L) in the rat dentate gyrus (A–D), CA3 (E–H), and CA1 (I–L)**. Results are summarized in a rating gray-scale of the immunoreactivity in somata and fibers of each layer and stratum of the hippocampus **(M)**. Three labeling intensities are represented from “f/s” meaning “low” fiber and/or somata (above the background density) to “fff/sss” meaning “high” fiber and/or somata (according to the highest signal density in the specimen) or without immunoreactivity (–). Scale bars are indicated in each image. Abbreviations: alv, alveus; DG, dentate gyrus; gcl, granular cell layer; ml, molecular layer; pcl, polymorphic cell layer (hilus); SL, stratum lucidum; SL-M, stratum lacunosum-moleculare; SO, stratum oriens; SP, stratum pyramidale; SR, stratum radiatum.

To address the distribution of the immunohistochemical expression of CB_1_, DAGLα, MAGL, FAAH in the rat hippocampus, coronal sections of the rat hippocampus were subjected to immunohistochemical analysis (Figure 2). The distribution of the immunohistochemical expression of the CaBPs calbindin, calretinin, and parvalbumin in the rat hippocampus is shown in the Figure S1. The results of this analysis are described in the main and supplementary texts and summarized in a rating scale included in Figure 2 and Figure S1. Gray-scale values measured in the dentate gyrus, CA3 and CA1 (Figure 2M and Figure S1J) are represented on an arbitrary scale of three labeling intensities, from “f/s” meaning “low” fiber and/or somata (above the background density) to “fff/sss” meaning “high” fiber and/or somata (according to the highest signal density in the specimen).

### Dentate gyrus

Intense CB_1_ immunoreactivity in the dentate gyrus was associated with a dense network of fibers in the molecular layer, being more prominent in its inner part adjacent to the granular cell layer (Figure 2A). Most of these CB_1_ immunoreactive (CB^+^_1_) fibers in the inner part of the molecular layer may correspond to the hilar mossy cell fiber terminals, whereas those fibers in the outer parts may represent projections from the layer II of entorhinal cortex. We also observed numerous fibers in the polymorphic cell layer and in the granular cell layer. Fine CB^+^_1_ fibers showed numerous varicosities and terminals that surrounded and defined cell bodies, being evident in the granular cell layer (inset in Figure 2A). The intense DAGLα immunoreactivity in the dentate gyrus was mainly associated with a dense neuropil that was particularly prominent in the molecular layer and in the inner portion of the granular cell layer (Figure 2B). The neuropil may represent the apical and basal dendritic field of the granular cells. Weak staining was also observed in the granular and polymorphic cell layers (inset in Figure 2B). The dentate gyrus was characterized by the lack of MAGL immunoreactivity. Only scattered, weakly stained polymorphic cells can be observed (Figure 2C, inset). FAAH immunoreactivity was weakly detected in the somata of the granular cells, but was more evident in a number somata of polymorphic cells (Figure 2D, inset).

### Hippocampal CA fields

A dense network of fibers intensely stained for CB_1_ characterized the stratum pyramidale (SP) and two adjacent sublayers into the strata radiatum (SR) and oriens (SO) from the CA3 to CA1 fields (Figures 2E,I). So, the sublayer of CB^+^_1_ fibers in the SR of CA3 defined the stratum lucidum (SL) and consisted of projections from the granular cells of the dentate gyrus that innervated the proximal part of the apical pyramidal dendrites. The remaining part of the SR also showed numerous fibers in contrast to the low number of fibers in the SO. It should be noted a well-defined band of CB^+^_1_ fibers that were observed along the boundary between the SR and the stratum lacunosum-moleculare (SL-M) (Figure 2I). Most of these fibers may represent Schaffer collaterals and commissural fibers, but possibly also direct axons from the layer III of the entorhinal cortex. Most CB^+^_1_ fibers contained numerous varicosities and terminals that mainly surrounded unstained pyramidal cell bodies and dendrites (inset in Figure 2I), but also defined cell profiles in the SR and SO (inset in Figure 2E). We could not observe CB^+^_1_ fibers in the alveus. In the hippocampal CA fields, intense DAGLα^+^ neuropil characterized the SO, SR, and SL-M, whereas the SP and the boundary between the SR and SL-M showed low immunostaining for DAGLα (Figures 2F,J). Most of the neuropil may represent the dendritic field of the pyramidal neurons, being more prominent in the SO, that is, in its basal dendritic tree. An intense network of MAGL^+^ fibers (possibly axon terminals) characterized the hippocampal CA fields, being more pronounced in the SR and SO from CA3 to CA1 (Figures 2G,K). Interestingly, numerous MAGL-containing pericellular basket terminals surrounded numerous unstained pyramidal cell somata, mostly in the CA3 field (inset in Figure 2G). The SL-M and the alveus showed very weak staining for MAGL (Figure 2K). FAAH immunoreactivity was intensely detected in the somata and proximal dendrites of the pyramidal neurons from CA3 to CA1 (Figures 2H,L). FAAH^+^ neurons were also observed in the SR and SO, as well as small cells in the alveus (insets in Figure 2L).

### Co-localization of CB1 and CaBPs in the adult rat hippocampus

**Figure 3:**
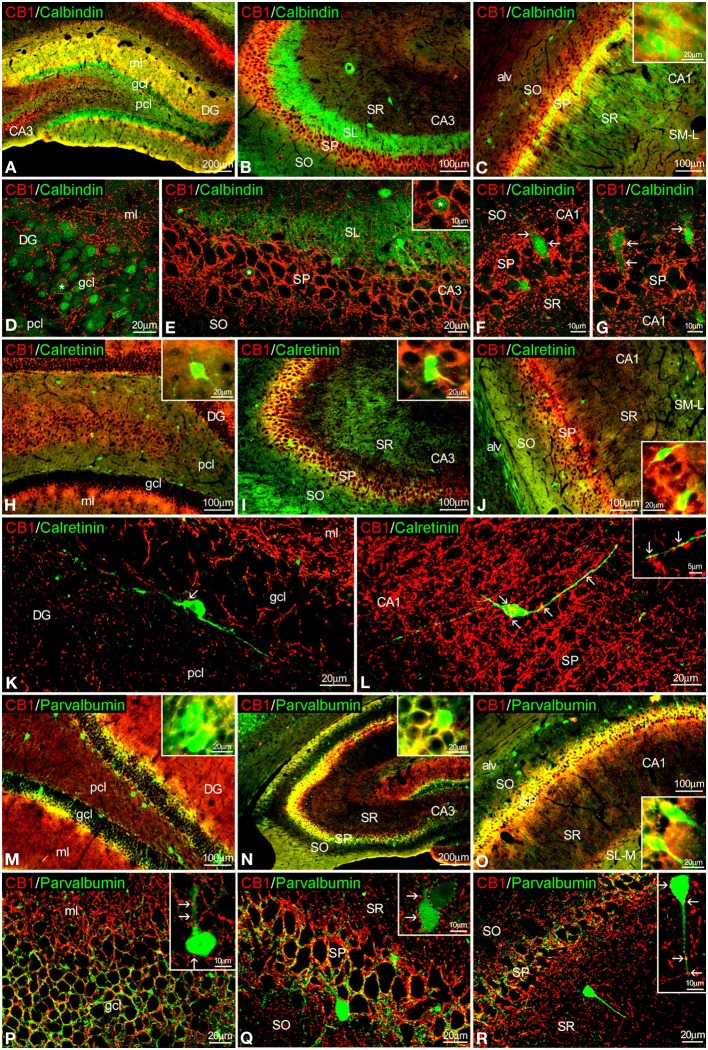
**Co-localization of CB_1_ and calbindin, calretinin, or parvalbumin in the rat hippocampus**. Low resolution epifluorescence photomicrographs **(A–C,H–J,M–O)** and high resolution confocal laser scanning photomicrographs **(D–G,J–L,P–R)** showing labeling for calbindin, calretinin, or parvalbumin (green) and CB_1_ (red) in dentate gyrus, CA3, and CA1 areas. Arrows indicate CB_1_ expression in fiber terminals surrounding cells expressing calbindin, calretinin, and parvalbumin. **(L)** Image showing the overlap of a Z-serie with a size-depth of 14.1 μm. For abbreviations see Figure 2. Scale bars are indicated in each image.

To study the co-expression of CB1 and the CaBPs calbindin D28k, calretinin, and parvalbumin in the hippocampus, coronal sections were subjected to double-immunolabeling and confocal microscope analysis. Calbindin was highly expressed in most granular cells of the dentate gyrus, few cells of SP and SR of CA3 and a high number of cells in the SP and SO of CA1 (Figures 3A–G). Thus, the intense network of CB1 immunofluorescent fibers were closely surrounding the calbindin^+^ cells localized in the granular cell layer of the dentate gyrus and the SP of CA3 and CA1 fields (Figures 3D–G). In the SP of CA1, CB1^+^ fibers were localized around calbindin^+^ cell bodies and proximal axons in a basket-like manner (Figures 3F,G). CB1^+^ fibers and terminals were also observed within the intense calbindin^+^ neuropil of the SL of CA3 (Figure 3E).

Regarding calretinin, we could observe that few CB1^+^ terminals were closely attached to the surface of calretinin^+^ cells localized in the inner border of the granular cell layer of the dentate gyrus and the SP of CA3 and CA1 (Figures 3H–L).

Interestingly, both CB1 and parvalbumin immunofluorescences were localized showing an intercalated meshwork of terminals, mostly around unstained principal cell profiles, in the granular cell layer of the dentate gyrus and the SP of CA3 and CA1 (Figures 3M–R). Thus, some CB1^+^ terminals were also attached to a number of parvalbumin^+^ cells localized between the granular and polymorphic cell layers of the dentate gyrus, the SP of CA3 and the SR of CA1 (Figures 3P–R, insets).

### Co-localization of DAGLα and CaBPs in the adult rat hippocampus

**Figure 4:**
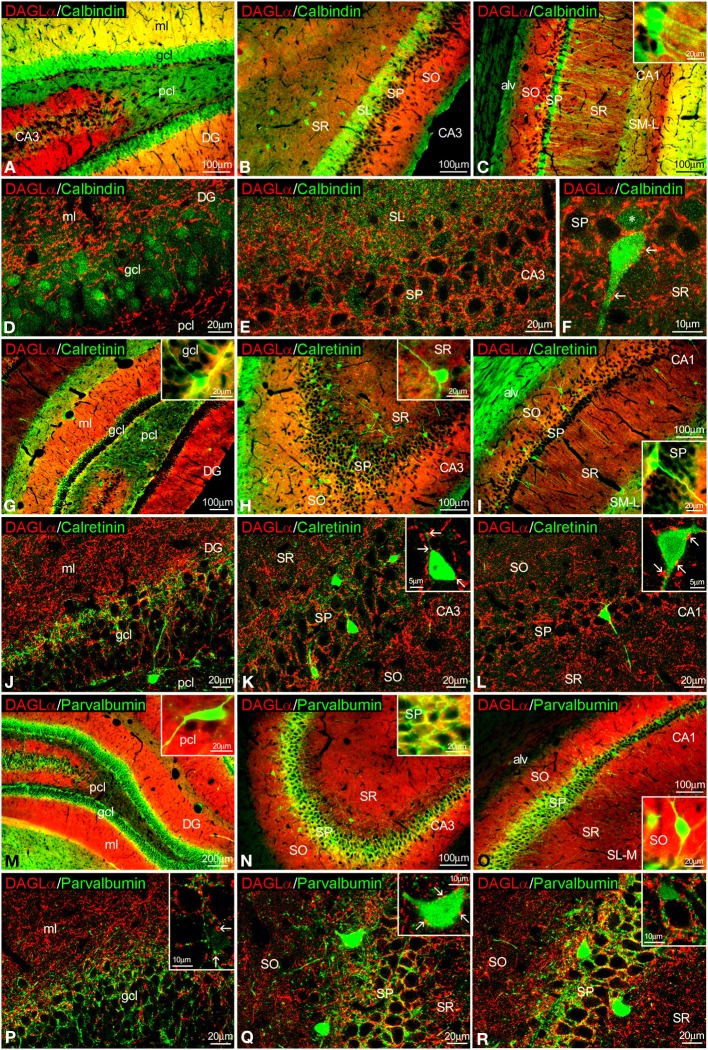
**Co-localization of DAGLα and calbindin, calretinin, or parvalbumin in the rat hippocampus**. Low resolution epifluorescence photomicrographs **(A–C,G–I,M–O)** and high resolution confocal laser scanning photomicrographs **(D–F,J–L,P–R)** showing labeling for calbindin, calretinin, or parvalbumin (green) and DAGLα (red) in dentate gyrus, CA3, and CA1 areas. Arrows indicate DAGLα process labeling around cells expressing calbindin, calretinin, and parvalbumin. For abbreviations see Figure 2. Scale bars are indicated in each image.

To study the co-expression of DAGLα and the CaBPs calbindin D28k, calretinin, and parvalbumin in the hippocampus, coronal sections were also subjected to double-immunolabeling and confocal microscope analysis. A high number of calbindin^+^ cells localized in the granular cell layer of the dentate gyrus and the SP of CA1 were closely surrounded by DAGLα^+^ neuropil and processes (Figures 4A–F, inset). On the other hand, we couldn't find DAGLα^+^ neuropil surrounding calbindin^+^ cells in the SP of CA3 (Figure 4E). DAGLα^+^ processes were also observed within the intense calbindin^+^ neuropil of the SL of CA3 (Figure 4E).

We observed DAGLα^+^ neuropil and processes on the surface of the somata and proximal axons of calretinin^+^ cells localized in the granular cell layer of the dentate gyrus and the SP of CA1/3 fields (Figures 4G–L, insets).

An intercalated network of processes and terminals showing immunofluorescence for DAGLα and parvalbumin, respectively, were found surrounding unstained principal cell profiles in the granular cell layer of the dentate gyrus and the SP of CA1/3 fields (Figures 4M–R). Some DAGLα^+^ processes were also observed close to a number of parvalbumin^+^ cells localized in the SP of CA1/3 fields (Figure 4Q, inset).

### Co-localization of MAGL and CaBPs in the adult rat hippocampus

**Figure 5:**
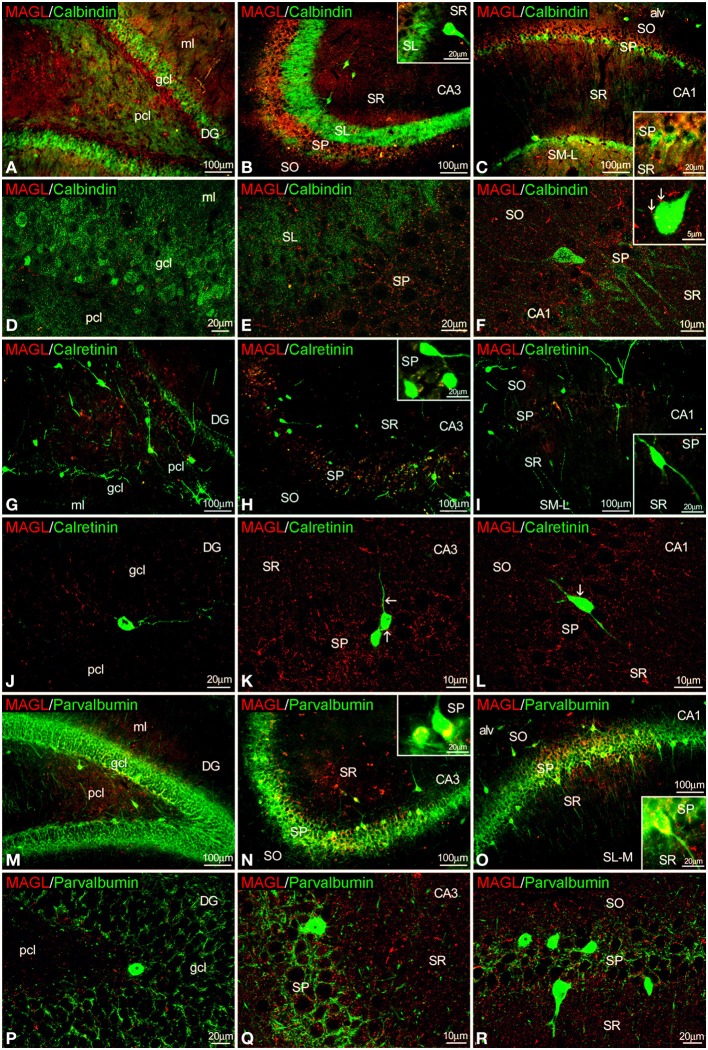
**Co-localization of MAGL and calbindin, calretinin, or parvalbumin in the rat hippocampus**. Low resolution epifluorescence photomicrographs **(A–C,G–I,M–O)** and high resolution confocal laser scanning photomicrographs **(D–F,J–L,P–R)** showing labeling for calbindin, calretinin, or parvalbumin (green) and MAGL (red) in dentate gyrus, CA3, and CA1 areas. Arrows indicate MAGL fiber terminals closely attached to cells expressing calbindin and calretinin, but not parvalbumin. For abbreviations see Figure 2. Scale bars are indicated in each image.

MAGL^+^ terminals were observed adjacent to some calbindin^+^ cells in SP of CA1, as well as unstained cell profiles, in the SP of CA3 and CA1(Figures 5A–F, inset). We couldn't find MAGL labeling surrounding calbindin^+^ cells in the granular cell layer of the dentate gyrus (Figure 5D). MAGL^+^ terminals were also found on the surface of few calretinin^+^ cells observed in the SP of CA1/3, but not in the dentate gyrus (Figures 5G–L). MAGL^+^ terminals were found intercalated into the meshwork of parvalbumin^+^ terminals, mostly close to unstained principal cell profiles, in the SP of CA3 and CA1 but not in the dentate gyrus (Figures 5M–R). No expression of MAGL was found in the parvalbumin^+^ cells.

### Co-localization of FAAH and CaBPs in the adult rat hippocampus

**Figure 6:**
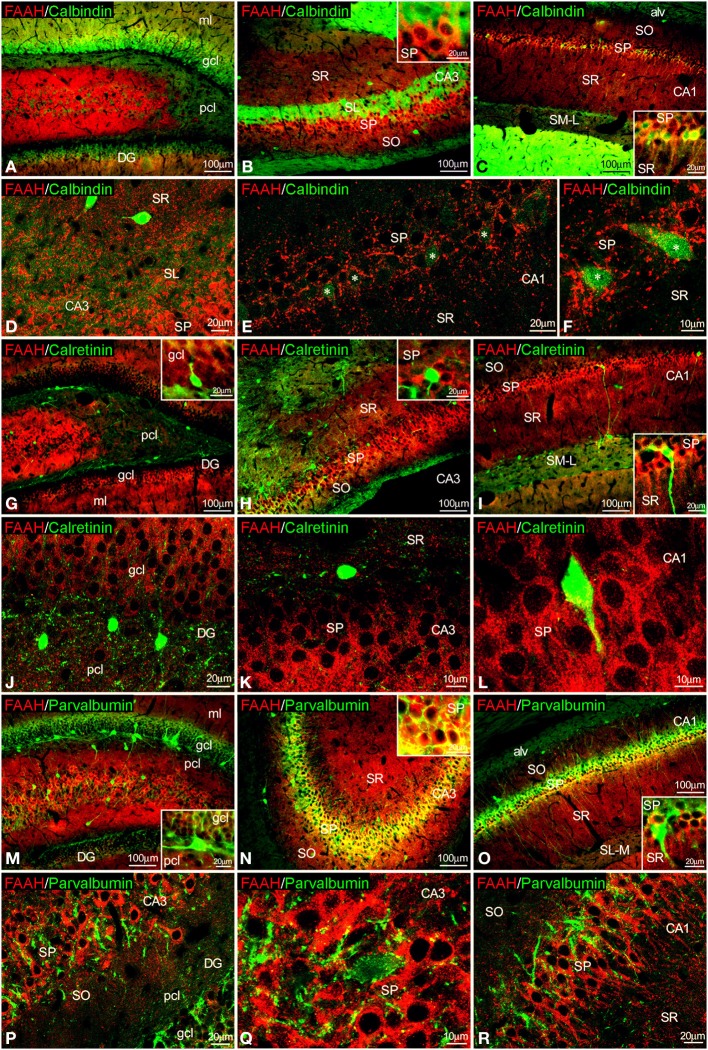
**Co-localization of FAAH and calbindin, calretinin or parvalbumin in the rat hippocampus**. Low resolution epifluorescence photomicrographs **(A–C,G–I,M–O)** and high resolution confocal laser scanning photomicrographs **(D–F,J–L,P–R)** showing labeling for calbindin, calretinin, or parvalbumin (green) and MAGL (red) in dentate gyrus, CA3, and CA1 areas. Asterisks indicate FAAH expression in calbindin^+^ pyramidal cells localized specifically in CA1. For abbreviations see Figure 2. Scale bars are indicated in each image.

FAAH/calbindin co-expression was neither detected in the dentate gyrus nor in the CA3 (Figures 6A,B,D). All calbindin^+^ cells detected in the SP of CA1 showed FAAH expression (Figures 6C,E,F, insets). FAAH labeling was specifically localized in the inner surface of somata and the proximal axons of the calbindin^+^ cells (Figure 6F). FAAH and calretinin (Figures 6G–L) or FAAH and parvalbumin (Figures 6M–R) co-expressions have not been found in the hippocampus. However, parvalbumin^+^ fibers and terminals were observed around FAAH^+^ cells in the principal cell layers of CA1/3 fields (Figures 6Q,R).

### Analysis of orthogonal sectioning

**Figure 7:**
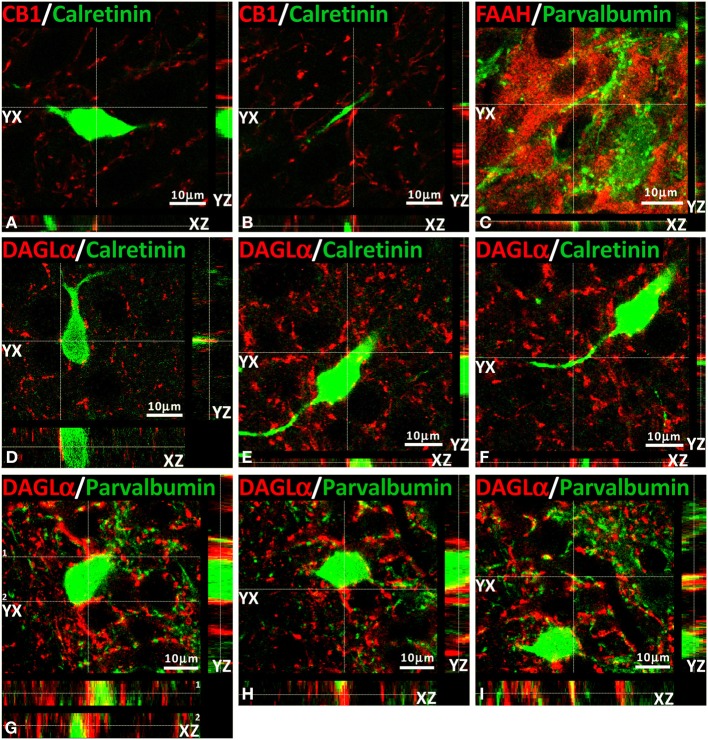
**Orthogonal sectioning views**. Selected z-scanning series of images showing double immunofluorescence (red and green) of CB1, FAAH, DAGLα, calretinin, and parvalbumin. To analyze the labeling from different orientations, it is represented the three planes of view for one point, as was indicated by the crossed dashed lines (YX plane, YZ plane, and YX plane).

We have also conducted several z-scanning series and represented orthogonal sectioning views to analyze selected labeling from different orientations (Figure 7). As a consequence, we can get a better appraisal of the double immunofluorescence when the co-localization is doubtful. Thus, we selected several orthogonal representations of z-series for CB_1_, FAAH, DAGLα, calretinin, and parvalbumin showing the three planes of view for one point, as was indicated by the crossed dashed lines in Figure 7 (YX plane, YZ plane, and YX plane). We can observe in two planes of each orthogonal representation that both labeling were adjacent, suggesting a closely approximation of the two proteins analyzed and confirming the results previously described for each double immunofluorescence.

## Discussion

**Figure 8:**
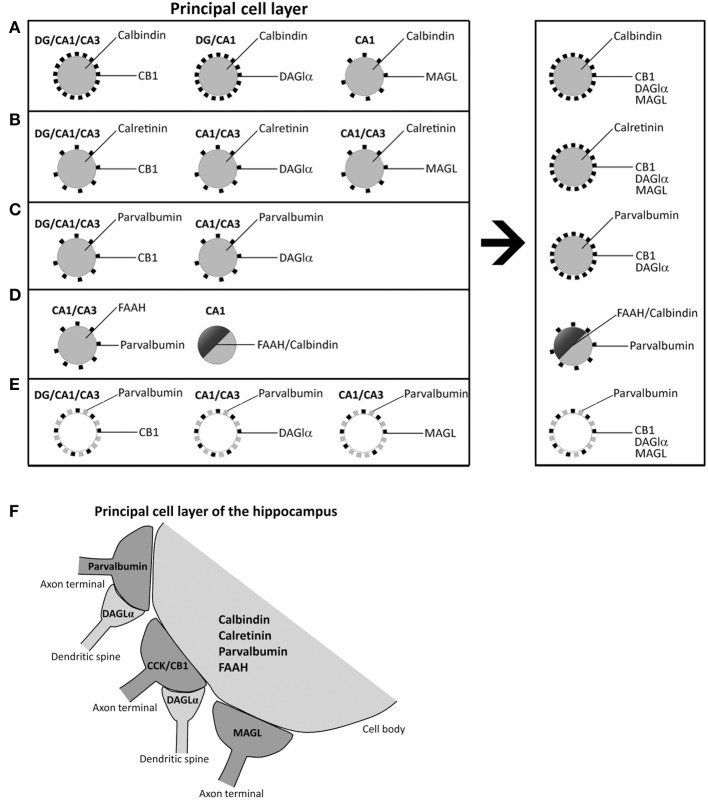
**Schematic representation that hypothesizes the neuronal substrates containing 2-AG/CB1 signaling system and the CaBPs calbindin, calretinin, and parvalbumin in the different hippocampal regions. (A)** Calbindin^+^ neurons in principal cell layers were closely surrounded by CB^+^_1_, DAGLα^+^, and MAGL^+^ terminals and processes. **(B)** Hippocampal cells containing calretinin were closely surrounded by CB^+^_1_, DAGLα^+^, and MAGL^+^ terminals and processes. **(C)** Hippocampal cells containing parvalbumin were closely surrounded by processes expressing CB_1_ and DAGLα, but not MAGL. **(D)** A number of hippocampal pyramidal cells co-expressed both calbindin and FAAH in CA1, which could be surrounded by parvalbumin^+^ terminals. **(E)** Fibers and puncta expressing parvalbumin and CB_1_, DAGLα, or MAGL were arranged in an intercalated manner around cells of the principal cell layers. **(F)** A Scheme that summarizes the putative anatomical organization of the neuronal substrates that express 2-AG/CB_1_ signaling system-related molecules associated with the CaBPs.

The role of the endocannabinoid 2-AG on the retrograde suppression of excitatory/inhibitory synaptic transmission requires both release from membrane precursors by the Ca^2+^-dependent DAGLα and the activation of CB_1_ receptors that modulate Ca^2+^-dependent mechanisms such as the inhibition of neurotransmitter release (D'Amico et al., 2004; Di et al., 2005; Katona and Freund, 2008). Thus, the identification of specific 2-AG/CB1signaling system-containing neural substrates that express selective Ca^2+^-binding proteins (CaBPs) should be considered when analyzing the functional significance of the cannabinoid signaling associated with calcium handling and hippocampal function. Our study provides evidence for an anatomical distribution of 2-AG/CB_1_ signaling system in the hippocampus, by identifying the localization and co-expression of CB_1_, DAGLα, MAGL, and FAAH and the CaBPs calbindin, calretinin, and parvalbumin that have not been previously described. Due to the specific localization of CB_1_ and MAGL in axon terminals, DAGLα in dendritic spines, FAAH in somata and dendrites, and CaBPs in certain cell bodies and fibers, we were able to describe at least four neural networks, which are differentially distributed in the hippocampus (Figure 8). However, it should be also noted that there are some exceptions in a regional-dependent manner: (1) CB1^+^ terminals, DAGLα^+^ neuropil, and MAGL^+^ terminals were tightly attached to calbindin^+^ neurons in the principal cell layers (Figure 8A). We couldn't detect DAGLα^+^ processes surrounding calbindin^+^ cells in CA3 and MAGL^+^ terminals surrounding calbindin^+^ cells in the dentate gyrus and CA3. (2) We observed that hippocampal cells containing calretinin were closely surrounded by CB^+^_1_ terminals, DAGLα^+^ neuropil, and MAGL^+^ terminals. However, we couldn't find DAGLα^+^ processes and MAGL^+^ terminals surrounding calretinin^+^ cells in the dentate gyrus (Figure 8B). (3) We also found that hippocampal cells containing parvalbumin were clearly surrounded by terminals and processes expressing CB_1_ and DAGLα, but not MAGL. We couldn't find DAGLα^+^ processes surrounding parvalbumin^+^ cells in the dentate gyrus (Figure 8C). (4) A number of hippocampal pyramidal cells co-expressed both calbindin and FAAH in CA1, which could be innervated by parvalbumin^+^ terminals (Figure 8D). We didn't observe FAAH expression in calbindin^+^ cells found in dentate gyrus and CA3. Regarding these results, we observed that terminals and processes that expressed parvalbumin and CB_1_, DAGLα, or MAGL were tightly attached in an intercalated manner around cells of the principal cell layers (Figure 8E). Finally, Figure 8F shows a scheme that summarizes the putative anatomical organization of the structures that express 2-AG/CB_1_ signaling system-related molecules associated with the CaBPs.

Our results confirm data from previous studies on the presence and localization of CB_1_, DAGLα, MAGL, and FAAH (Tsou et al., 1998a,b; Egertová and Elphick, 2000; Egertová et al., 2003; Gulyás et al., 2004; Yoshida et al., 2006; Suárez et al., 2009), as well as the CaBPs calbindin, calretinin, and parvalbumin, in the rodent hippocampus (Baimbridge and Miller, 1982; Kosaka et al., 1987; Gulyás et al., 1992; Miettinen et al., 1992). Thus, it has been well-documented that CB_1_ and MAGL labeling constituted axon terminals, DAGLα labeling represented dendritic spines and FAAH was present in somata and dendrites of principal cells (Egertová and Elphick, 2000; Gulyás et al., 2004; Yoshida et al., 2006). Calbindin immunoreactivity was described in the granular cells of the dentate gyrus, pyramidal cells of CA1 and scattered interneurons of the hippocampal SR (Baimbridge and Miller, 1982). Calretinin immunoreactivity was almost exclusively described in the GABAergic non-pyramidal spiny-free cells in all layers of the dentate gyrus and the CA1/3 fields, being most abundant in the polymorphic cell layer of the dentate gyrus (hilus). Interestingly, it has been also described the existence of GABA-negative calretinin-containing spiny neurons that were specifically localized in the polymorphic cell layer of the dentate gyrus and the SL of CA3 (Gulyás et al., 1992; Miettinen et al., 1992). Parvalbumin^+^ cells were specifically localized in the granular and polymorphic cell layers of the dentate gyrus and the strata oriens and pyramidale in CA1/3 fields of the rat hippocampus (Kosaka et al., 1987). They have been considered a subpopulation of GABAergic interneurons, including basket and axo-axonic cell types, which innervate the somata and proximal axons of pyramidal cells, respectively (Soriano et al., 1990).

In the present study, we observed that calbindin^+^, calretinin^+^, and parvalbumin^+^ cells were tightly attached to CB^+^_1_ fiber terminals. Most CB^+^_1_ terminals surrounding the somata and proximal dendrites of pyramidal neurons were cholecystokinin^+^ (CCK) GABAergic interneurons (basket cells) and, to a lower extent, calbindin D-28k^+^ GABAergic interneurons (Katona et al., 1999; Marsicano and Lutz, 1999; Tsou et al., 1999). However, parvalbumin^+^ GABAergic interneuron terminals localized in pyramidal cell layers were negative for CB_1_ (Katona et al., 1999; Marsicano and Lutz, 1999). Regarding our results, we can suggest that most fiber terminals containing CB_1_/CCK probably innervate both calbindin^+^ pyramidal neurons (probably including calbindin^+^/FAAH^+^ pyramidal neurons in CA1) and calretinin^+^ and parvalbumin^+^ interneurons localized in the principal layers of the hippocampus (Gulyás et al., 1991; Marsicano and Lutz, 1999). Moreover, parvalbumin^+^ fiber terminals, which represent another GABAergic interneuron type, could specifically innervate FAAH^+^/calbindin^−^ pyramidal neurons in CA3. Regarding the functional implication, it has been demonstrated that (1) parvalbumin^+^ CA1 interneurons are required for spatial working memory but not for spatial reference (Murray et al., 2011); and (2) the specific CB1 activation in the hippocampus impairs working tasks (Wise et al., 2009). So, it could be conceivable that the innervations of CB1^+^ fibers on parvalbumin^+^ interneurons and the lack of neurons with CB1/parvalbumin co-expression should be relevant in the functioning of the spatial memory.

It was described that DAGLα is accumulated in postsynaptic pyramidal spines, which are closely attached to CB_1_ inhibitory terminals (Yoshida et al., 2006). We observed that terminals and processes expressing parvalbumin and CB_1_, DAGLα, or MAGL were arranged in an intercalated manner around cells of the principal cell layers. Our results agree with the proximity between DAGLα^+^ processes and CB1^+^ terminals, but also suggested that DAGLα^+^ processes could also be tightly attached to MAGL and parvalbumin terminals. Thus, we can speculate that DAGLα^+^ processes could be associated to at least two types of GABAergic interneurons, CB1^+^/CCK^+^ inhibitory interneurons and parvalbumin^+^ inhibitory interneurons. It is reasonable to suggest that the tiny neuropil labeling for DAGLα observed in the non-principal cell layers can represent dendritic spines of granular and pyramidal cells. However, the localization of DAGLα^+^ neuropil closely circling calbindin^+^, calretinin^+^, and parvalbumin^+^ cells in the principal cell layers may suggest a different origin that must be investigated in future studies.

We observed that calbindin^+^ pyramidal cells expressed FAAH specifically in the CA1 field. It is possible that these cells can be specifically innervated by CB1^+^/CCK^+^ terminals. Since calcium influx is a requirement for the synthesis of endocannabinoids (Tsou et al., 1998b; Bisogno et al., 2003; Nyilas et al., 2008; Gao et al., 2010; Tanimura et al., 2010) and FAAH is associated with the membrane of cytoplasmic organelles known to store Ca^2+^ (Gulyás et al., 2004), the specific co-expression of the NAE-hydrolyzing enzyme FAAH and the calcium-binding protein calbindin in CA1 pyramidal cells also suggests the possibility of a functional relationship between the clearance of endocannabinoids and the buffering of intracellular calcium in the postsynaptic structures of these neurons.

In conclusion, our data showed that the identification of specific 2-AG/CB1 signaling system-containing neural substrates that expressed the CaBPs calbindin, calretinin, and parvalbumin provide a neuroanatomical framework that shed light to the identification of the cellular networks that can be involved in the functioning of the hippocampal endocannabinoid system.

## Author contributions

All authors had full access to all the data in the study and take responsibility for the integrity of the data and the accuracy of the data analysis. Study concept and design: Juan Suárez, Fernando Rodríguez de Fonseca Acquisition of data: Patricia Rivera, Sergio Arrabal, Antonia Serrano, Francisco J. Pavón, Antonio Vargas, Leticia Rubio, Jesús M. Grondona, Margarita Pérez-Martín, Manuel Cifuentes. Analysis and interpretation of data: Patricia Rivera, Antonia Serrano, Juan Suárez. Drafting of the manuscript: Patricia Rivera, Juan Suárez. Critical revision of the manuscript for important intellectual content, obtained funding and study supervision: Juan Suárez, Fernando Rodríguez de Fonseca.

### Conflict of interest statement

The authors declare that the research was conducted in the absence of any commercial or financial relationships that could be construed as a potential conflict of interest.
