# Supplementary material for: Localization of the cannabinoid CB1 receptor and the 2-AG synthesizing (DAGLα) and degrading (MAGL, FAAH) enzymes in cells expressing the Ca2+-binding proteins calbindin, calretinin, and parvalbumin in the adult rat hippocampus
Source: Front Neuroanat. 2014 Jun 27;8:56. doi: 10.3389/fnana.2014.00056 (PMC4073216; doi:10.3389/fnana.2014.00056)
Supplement: Supplementary file 1 [file DataSheet1.PDF]

SUPPLEMENTARY RESULTS

Distribution of CaBPs in the adult rat hippocampus

To address the distribution of the immunohistochemical expression of the CaBPs calbindin D28k, calretinin and parvalbumin in the rat hippocampus, coronal sections of the rat hippocampus were subjected to immunohistochemical analysis (Fig. S1). The results of this analysis are also summarized in a rating scale included in Figure S1 (dentate gyrus and CA fields, respectively). Gray-scale values measured in the dentate gyrus and the CA3 and CA1 fields are represented on an arbitrary scale of three labeling intensities, from "f/s" meaning "low" fiber and/or somata (above the background density) to "fff/sss" meaning "high" fiber and/or somata (according to the highest signal density in the specimen).

Dentate gyrus

A high number of immunoreactive cells for calbindin D28k was detected in the granular cell layer of dentate gyrus (Fig. S1A). A high density of fibers was intensely stained for calbindin in the molecular layer of the dentate gyrus. Weaker staining for calbindin was observed in fibers of the polymorphic cell layer of the dentate gyrus. Intense immunoreactivity for calretinin was associated with the somata and proximal projections of a discrete number of cells localized in the polymorphic cell layer of the dentate gyrus (Fig. S1B). A weak network of calretinin$^+$ fibers was detected in the border between the granular cell layer and molecular layer of the dentate gyrus (Fig. S1F, inset). A discrete number of intensely stained cells for parvalbumin, comprising somata and proximal projections, was observed in the granular cell layer of the dentate gyrus (Fig. S1C, inset). Parvalbumin immunoreactivity was also associated with a dense

meshwork of fibers localized in the granular cell layer of the dentate gyrus. These fibers were arranged surrounding unstained profiles of the granular cells (Fig. S1C, inset).

Hippocampal CA fields

Calbindin$^+$ cells and fibers were observed in the SL and SR of CA3 (Fig. S1D, inset) and SP and SR of CA1 (Fig. S1G, inset). There appeared to be a slightly higher number of stained cells for calbindin in the more temporal aspect of CA1. Weaker staining for calbindin was also observed in fibers situated in the limit between the SR and SL-M of CA1 (Fig. S1G). Calretinin immunoreactivity was associated with the somata and large projections of a discrete number of cells localized in all strata of CA1/3 fields (Figs. S1E, H, insets). Intensely stained cells for parvalbumin were observed in the SP of CA3 (Fig. S1F, inset) and SP and SO of CA1 (Fig. S1I, inset). Parvalbumin immunoreactivity was also associated with a dense meshwork of fibers localized in the SP of CA1/3 fields. These fibers were arranged surrounding unstained profiles of the principal cells (Figs. S1F, S1I, insets).

Figure S1. Immunohistochemical expression of the CaBPs calbindin (A, D, G), calretinin (B, E, H) and parvalbumin (C, F, I) in the rat dentate gyrus (A-C), CA3 (D-F) and CA1 (G-I). Results are summarized in a rating gray-scale of the immunoreactivity in somata and fibers of each layer and stratum of the hippocampus (J). Three labeling intensities are represented from “f/s” meaning “low” fiber and/or somata (above the background density) to “fff/sss” meaning “high” fiber and/or somata (according to the highest signal density in the specimen) or without immunoreactivity (-). Scale bars are indicated in each image. Abbreviations: DG, dentate gyrus; gcl, granular cell layer; ml, molecular layer; pcl, polymorphic cell layer (hilus), SL, stratum lucidum; SL-M,

stratum lacunosum-moleculare; SO, stratum oriens; SP, stratum pyramidale; SR, stratum radiatum.
