# Supplementary material for: Localization of the cannabinoid CB1 receptor and the 2-AG synthesizing (DAGLα) and degrading (MAGL, FAAH) enzymes in cells expressing the Ca2+-binding proteins calbindin, calretinin, and parvalbumin in the adult rat hippocampus
Source: Front Neuroanat. 2014 Jun 27;8:56. doi: 10.3389/fnana.2014.00056 (PMC4073216; doi:10.3389/fnana.2014.00056)
Supplement: Supplementary file 2 [file DataSheet2.PDF]

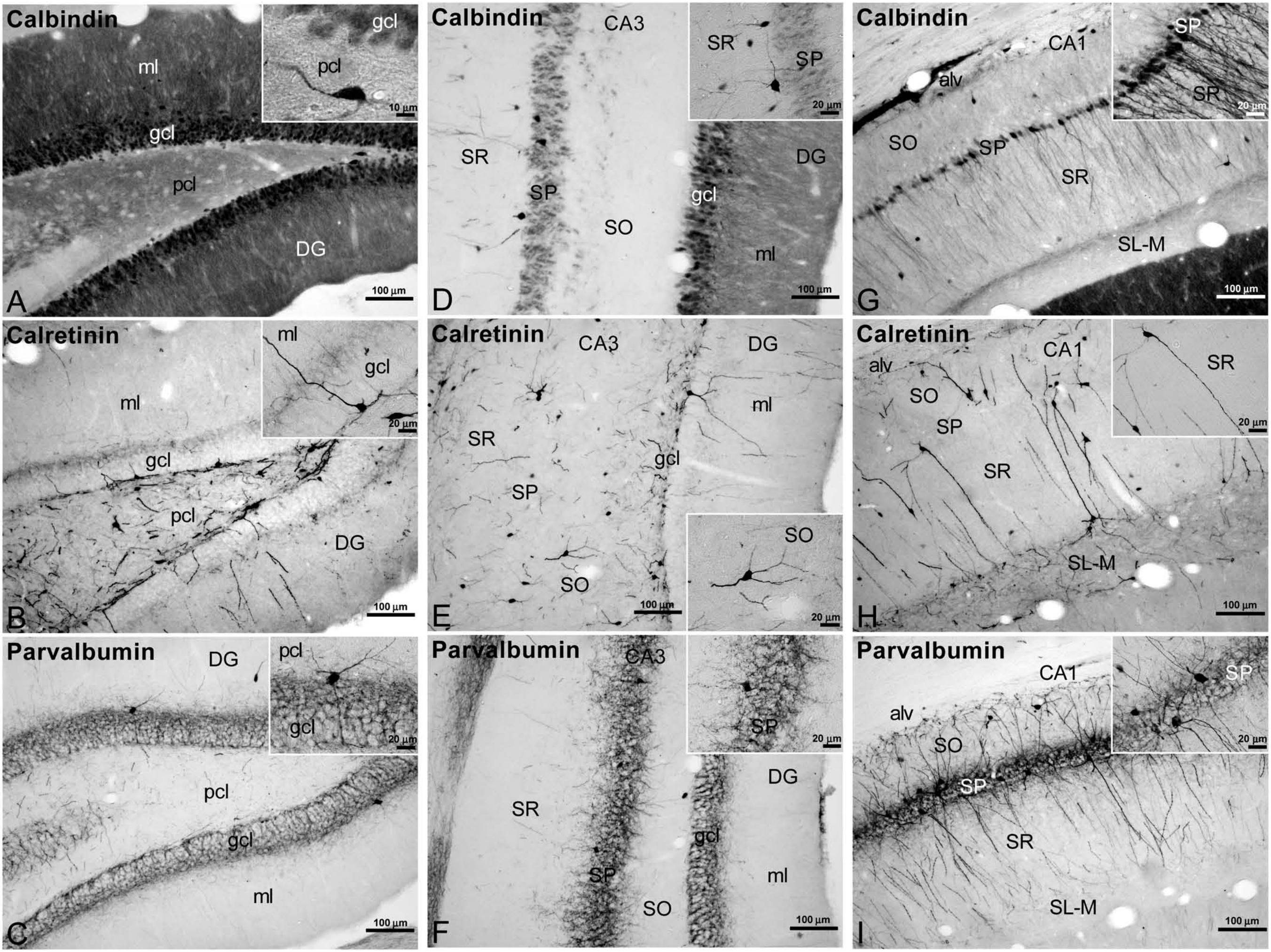

J

	Dentate gyrus			CA3 field			CA1 field			
	ml	gcl	pcl	SO	SP	SR	SO	SP	SR	SL-M
Calbindin	fff	sss	s/f	-	ss	-	s	ss	s/f	f
Calretinin	-	s/f	s	s	s	s	s	s	s	s/f
Parvalbumin	-	s/fff	f	-	s/ff	-	s/f	s/ff	f	-
